# The scientific journey of a novel adjuvant (AS37) from bench to bedside

**DOI:** 10.1038/s41541-024-00810-6

**Published:** 2024-02-08

**Authors:** Ugo D’Oro, Derek T. O’Hagan

**Affiliations:** 1 GSK, Siena, Italy; 2 GSK, Rockville, MD USA

**Keywords:** Adjuvants, Drug delivery

## Abstract

A decade ago, we described a new approach to discover next generation adjuvants, identifying small-molecule immune potentiators (SMIPs) as Toll-like receptor (TLR)7 agonists. We also optimally formulated these drugs through adsorption to aluminum salts (alum), allowing them to be evaluated with a range of established and early-stage vaccines. Early proof-of-concept studies showed that a TLR7 agonist (TLR7a)-based SMIP, when adsorbed to alum, could perform as an effective adjuvant for a variety of different antigens, in both small and large animals. Studies in rodents demonstrated that the adjuvant enhanced immunogenicity of a recombinant protein-based vaccine against *Staphylococcus aureus*, and also showed potential to improve existing vaccines against pertussis or meningococcal infection. Extensive evaluations showed that the adjuvant was effective in non-human primates (NHPs), exploiting a mechanism of action that was consistent across the different animal models. The adjuvant formulation (named AS37) has now been advanced into clinical evaluation. A systems biology-based evaluation of the phase I clinical data with a meningococcal C conjugate vaccine showed that the AS37-adjuvanted formulation had an acceptable safety profile, was potent, and activated the expected immune pathways in humans, which was consistent with observations from the NHP studies. In the intervening decade, several alternative TLR7 agonists have also emerged and advanced into clinical development, such as the alum adsorbed TLR7/8 SMIP present in a widely distributed COVID-19 vaccine. This review summarizes the research and early development of the new adjuvant AS37, with an emphasis on the steps taken to allow its progression into clinical evaluations.

## Synthetic TLR7A: an attractive and tractable target for drug discovery

Most of the recombinant proteins or purified subunit antigens used in human vaccines lack strong immunostimulatory properties. Therefore, vaccines comprising these antigens typically contain adjuvants, often the classical insoluble aluminum salts (alum) or sometimes more innovative approaches. The newer generation of adjuvants include a number of well defined multi-component Adjuvant Systems (AS). AS are currently used in several licensed vaccines, for example those protecting against respiratory syncytial virus (RSV), herpes zoster or malaria (AS01), (pre)pandemic influenza or SARS-CoV-2 vaccines (AS03), and vaccines against hepatitis B virus (HBV) or human papillomaviruses (AS04)^1–3^. In comparative adjuvant studies in humans, immune responses induced by the AS were typically stronger than those induced by alum, and resulted in increased and persistent induction of CD4^+^ T-helper (Th) cells, memory B cells (MBCs) and functional antibodies^4–9^. While alum mainly enhances antibody responses, next generation adjuvants are needed to potentiate and qualitatively tune both humoral and T-cell mediated immunity. Effective adjuvants are also needed to enable vaccine development for disease targets that are not yet vaccine preventable, such as HIV, HCV or HSV. In addition, for some diseases there is a lack of effective vaccines designed for infants, with a clear need to overcome reduced vaccine responsiveness in early childhood^10^.

By activating antigen presenting cells (APCs) such as dendritic cells (DCs), ligands of a class of innate immune receptors called Toll-Like Receptors (TLRs) serve as potent adjuvants^11,12^. Both AS01 and AS04 are TLR agonist (TLRa) containing adjuvants, both including the TLR4a MPL, while CpG ODN 1018, a TLR9a, has been included in several candidate SARS-CoV-2 vaccines, and is currently a component of a licensed HBV vaccine^13^. Furthermore, a TLR7/8a is a component of a licensed SARS-CoV-2 vaccine that received an emergency use authorization in India^14^. TLR7 is the receptor for guanosine- and uridine-rich sequences from single-stranded RNA present in RNA viruses^15^, and also recognizes bacterial RNA^16^. Unlike TLR8 expression which is defective in mice^17^, TLR7 expression is highly conserved across vertebrates^18^, allowing cross-species evaluations in the established animal models. In humans, this receptor can be found to some extent in monocytes, neutrophils, and B and T cells, but is ubiquitous in plasmacytoid (p)DCs. Via its adapter protein MyD88, TLR7a can activate DCs, which in turn drive Th1-type responses by enhancing NK cell-derived IFN-γ induction in the draining lymph nodes (dLN)^19^. TLR7/8-activated DCs have been detected following administration of these adjuvants in both adult and neonatal animal models^10^. In fact, given the often impaired Th1 responses in infants, this may be an interesting strategy to facilitate the development of improved pediatric vaccines^20^. Among the TLRa, only TLR7/8a induces the production of both type I IFN and IL-12 secreted by pDCs and conventional/myeloid DCs, respectively^21^. As these cytokines support CD8^+^ T-cell cross-priming, TLR7/8a-based adjuvants can potentially induce both Th1 cells and cytotoxic T lymphocytes^22^, which may be particularly beneficial for more challenging pathogens such as HIV.

Since their original discovery, chemically synthesized low molecular weight TLR7a compounds have increasingly gained traction as potential next generation adjuvants, due to their homogeneity compared to natural products and their ease of manufacture. A well studied class of small molecule immune potentiators (SMIPs) are the imidazoquinolines, notably imiquimod (a human/murine TLR7a) and resiquimod (a human TLR7/8a and murine TLR7a). Both these SMIPS have been evaluated directly as immunotherapeutics, e.g. topical imiquimod in combination therapy for leishmaniasis, and in a commercial treatment for malignant and viral skin disorders, but also as vaccine adjuvants^23,24^. These SMIPs, when topically applied or injected in mice, were effective adjuvants for experimental HPV, HIV, or malaria vaccines^25–27^, and pretreatment with the licensed imiquimod cream enhanced seroconversion rates following intradermal influenza immunization in a clinical evaluation^28^. However, the pharmacokinetic/pharmacodynamic (PK/PD) profiles of injected low molecular weight molecules are typically characterized by rapid dissemination from the injection site and subsequent systemic circulation. This is followed by broad systemic immune activation and release of proinflammatory cytokines (e.g., IL-6, TNF-α, IFN-α/β), which is associated with substantial local and systemic reactogenicity^29^.

**Fig. 1:**
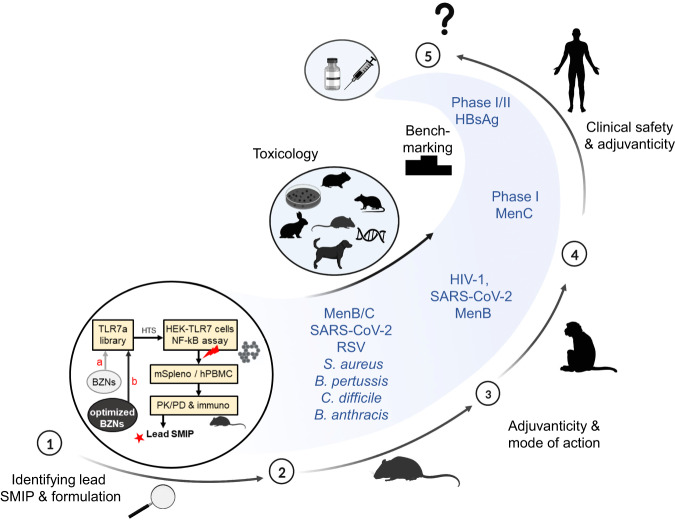
Journey from bench to bedside. Schematic summarizing the rational steps in the research and early development of AS37. (1) **a** High-throughput screening (HTS) of a library of chemical analogs of benzonaphthyridines (BZNs, a class of TLR7-active compounds) was initially performed to identify lead candidates^30^. Screening was initially performed in vitro, first based on ‘hits’ obtained using TLR7-expressing HEK293 (HEK-TLR7) cells in a NF-κB-dependent assay, then in murine splenocyte and human PBMC-based assays, followed by in vivo screening for favorable PK/PD and adjuvanticity in mouse models. However, the low solubility of the first-generation SMIPs caused overlong local half-lives and manufacturing/formulation challenges. (1) **b** A second approach aimed to achieve reduced localization of modified BZNs through efficient adsorption onto alum hydroxide. The secondary screening libraries contained soluble BZNs with linked phosphonates that could be adsorbed to alum via ligand exchange, with stable adsorption and higher solubility at neutral pH as key criteria for advancement to in vivo testing. Applicability to platform use of the alum-bound lead candidate (‘SMIP.7-10’) was confirmed by ultraperformance liquid chromatography, flow cytometry, confocal microscopy, phosphophilicity assays, zeta potential measurements, nuclear magnetic resonance, and mass/Raman spectroscopy^40^. Next, the adjuvant was developed in vivo in immunogenicity studies using mouse (**2**) and non-human primate (**3**) infectious disease models^42–44,46,51,52^, in parallel with in vitro and in vivo toxicology studies in various assays and animal models. Early clinical development (**4**) of the novel adjuvant system (‘AS37’) was performed in the context of meningitis serotype C (MenC) vaccines^54^, and in an ongoing comparative adjuvant study (NCT05561673), the outcomes of which will define its further clinical development (**5**). Created with BioRender.com.

Enlightened by the unraveling of the mechanisms of TLR7 activation, the rational design of alternative TLR7-targeting SMIPs was initiated^30^, aiming to exploit structural features and physicochemical factors that are associated with reduced toxicity and enhanced performance. This review summarizes the research and early development of a new TLR7-based adjuvant, now called AS37, and clarifies the journey traveled over the last decade to allow its progress into human evaluations (Fig. 1). We believe that this work represents the first time that a new adjuvant approach has been designed and developed based on utilizing broadly available tools, including high throughput screening and structure/function rationalization, along with sophisticated formulation science approaches.

## Nonclinical development of alum/TLR7a

### Discovery of potent TLR7a

Working on the assumption that enhanced muscle/dLN accumulation would induce the minimal localized inflammation levels and allow for optimal adjuvanticity, while eliminating the risk of systemic activation, Wu et al. discovered novel TLR7a SMIPs^30^ (Fig. 1a). They used high-throughput screening (HTS) and chemical design approaches to create novel compound libraries, and a class of TLR7-dependent SMIPs, the benzonaphthyridines (BZNs), were selected for chemical optimization. A key requirement of the HTS screening was the availability of assays to predict in vivo adjuvanticity, including the secretion of proinflammatory cytokines by TLR7-mediated activation of the NF-κB pathway. A TLR7-expressing HEK293 cell line enabled the detection of TLR7 activity and specificity, by monitoring the expression of a NF-κB-reporter gene. Hence, cell-based assays of TLR7-selective SMIP activity were performed to detect innate activation in splenocytes from wild type and TLR7-knock out mice, and human PBMCs. This was followed by a lipophilicity screen (based on polar surface area and partition coefficient/cLogP values) to provide preliminary evaluations of systemic distribution, which would be associated with potential toxicity. In addition, PK/PD profiles of identified candidates were assessed using IL-6 as a marker of likely systemic inflammation, which allowed a ranking of lead molecules based on their biodistribution and potency in mice. Of note, some BZN compounds were designed to be more lipophilic than resiquimod (the hydrophilic benchmark), in order to prolong muscle retention and avoid systemic distribution. This approach was undertaken to test the hypothesis that these PK/PD features would translate into improved adjuvanticity with reduced systemic reactogenicity.

The adjuvant effect of SMIPs after i.m. injection in mice was initially evaluated with a model antigen (ovalbumin), and then with *Neisseria meningitidis* serotype B (MenB) protein antigens from a licensed MenB vaccine^30^. Compared with resiquimod, type I IFN-responsive gene expression (as a marker of systemic inflammation) was significantly reduced in these experiments, while the injection-site half-life and potency in enhancing both IgG2a titers (which indicate Th1-skewed immunity) and MenB-functional serum bactericidal assay (SBA) titers, were all improved. However, local retention of the lipophilic BZNs alone was unreasonably long (>2 weeks), meaning that these compounds would be present (and thus potentially active) long after the key adjuvant effect (i.e., innate immune stimulation) had been achieved. Indeed, early research of the kinetics of successful TLRa-based adjuvants in mice had shown a transient innate immune response which peaked within the first 3 days post-injection^31,32^, and the (alum-formulated) TLR7a was later shown to display similar kinetics in NHPs and humans^33,34^, as detailed below. Furthermore, the insolubility of some BZN candidates could lead to drug precipitation, which is incompatible with practical formulations of aqueous vaccines. The BZN properties were therefore fine-tuned to better control muscle retention time and to optimize adjuvanticity, while allowing easy manufacturing and robust formulation scale-up.

### The path to clinical evaluation: chemical modification to create alum/TLR7a

It is generally recognized that sustained antigen adjuvant colocalization, which supports APC recruitment and activation, represents a key role for an effective ‘antigen delivery system’^13^. However, with regard to alum, the contribution of a local antigen depot to its mechanism of action remains unclear^33,35^. Although for the delivery of TLR7a SMIPs, various alternative approaches to alum have been evaluated^29^, adsorption to aluminum hydroxide was selected for clinical advancement of the BZNs. Indeed, while alum alone is less effective in enhancing Th1 responses (due to its inhibition of secretion of IL-12, a driver of Th1 responses, by DCs^36^), it combines several features considered attractive for an ideal delivery system^13,37^. Alum is well tolerated and effective in a number of licensed vaccines, and has an extensive safety record obtained through its use in common childhood vaccines over many decades. Moreover, the adjuvant is broadly available, inexpensive, compatible with multiple antigen types, able to stabilize protein antigens, and amenable to scalable and reproducible formulations. Finally, alum/TLRa-adjuvanted vaccine manufacture is well established, since this approach was previously used for the successful development of an HPV vaccine adjuvanted with alum adsorbed MPL (i.e., AS04)^37^.

The strongest adsorption to alum is usually mediated through phosphate groups on proteins, utilizing a ligand exchange mechanism, with the surface hydroxyls on alum. Since BZN analogs inherently lacked such groups, they were chemically functionalized by adding polyethylene glycol (PEG) linkers and a terminal phosphonate group. This modification resulted in more soluble BZN compounds that strongly adsorbed to alum, in contrast to the imidazoquinolines, which were shown to remain free in solution after being combined with alum^38^. In fact, this ability to easily modify BZN compounds chemically in order to alter their physicochemical properties and render them more suitable for purpose, is one of the fundamental attractions of SMIP-based adjuvants, and is in marked contrast to the many natural products that have previously been developed as vaccine adjuvants.

Finally, the library of second generation BZNs was optimized for potency/safety (using similar approaches used for the first generation compounds; Fig. 1b), and engineered to allow robust scale-up and GMP manufacturing.

### Preliminary proof-of-concept in mice

A pre-clinical evaluation of the SMIP TLR7a was initially performed with an established bacterial vaccine, i.e., recombinant MenB protein antigens^30^, followed at a later stage by evaluations with a second licensed bacterial vaccine, using a MenC protein polysaccharide conjugate (see below). The highly prevalent MenB serogroup affects mainly young children worldwide, while MenC associated disease tends to occur both in adults and young children.

In the MenB model, evaluation in mice revealed higher serum bactericidal assay (SBA) titers (the regulatory approved marker of clinical efficacy) for alum adsorbed *vs* free second generation SMIPS^30^. Given the large number of circulating MenB strains, these studies also focused on the breadth of the SBA response, since adjuvants have been shown to enhance the breadth of the response to pathogens^13^. Compared with alum alone, the alum-adsorbed TLR7a adjuvant increased SBA titers by four fold, killing all of the 17 tested MenB strains^30^. Serum total IgG as well as IgG2a were also increased, and the latter was associated with an enhanced Th1 cellular response.

To further explore the adjuvant’s ability to induce fully functional antibodies, its utility for vaccines providing postexposure prophylaxis against biothreats was tested using the mouse *Bacillus anthracis* (anthrax toxin) model^30^. Compared to alum alone, a single immunization with the alum/TLR7 elicited increased antigen-specific T cell, B cell and toxin-neutralizing responses, and enhanced protection against lethal challenge in naïve recipients of the sera from immunized mice. Mediated by TLR7-expressing DCs and B cells, the improved antibody quality suggested stronger involvement of T follicular helper (Tfh) cells^30^, a Th-cell lineage specialized in regulating B cell proliferation and antibody affinity maturation in the germinal centers (GCs)^39^.

### Formulation development

Product development of new vaccines is very much facilitated by the creation of fully liquid formulations which can be stored at 2–8 °C, and distributed as single vials or pre-filled syringes. However, in the early clinical studies, the TLR7a adjuvant was evaluated at different doses, which were obtained by dilution with alum alone, to keep the alum dose constant and allow dose evaluations of the SMIP only. Hence, the vaccine antigen in a standard vial was reconstituted with the adjuvant just before administration. Dose uniformity evaluations had previously demonstrated the feasibility of both a “bedside” mixing approach using a single-dose adjuvant vial at the final concentration, and an approach in which a high-dose adjuvant is first diluted with plain alum. A set of analytical techniques was applied to confirm the dose uniformity of the alum/TLR7 during this process (i.e., ultraperformance liquid chromatography, flow cytometry, confocal microscopy, phosphophilicity assays, zeta potential measurements, nuclear magnetic resonance, and mass/Raman spectroscopy)^40^. These studies showed that the new adjuvant has a stability profile similar to the well-established alum. Hence, similar to alum, the alum/TLR7a is expected to allow fridge-stable liquid vaccines in various presentation forms (although the stability profile of any vaccine product would still need to be evaluated, as stability is antigen-dependent). These approaches highlighted the long term potential of the new adjuvant for product development, but also in the short term allowed clinical evaluations to proceed with liquid or lyophilized antigens (see legend Fig. 1). Along with detailed evaluations of the PK profile for the SMIP, confirming its extended retention at the injection site and its eventual clearance from the body^41^, these data allowed the clinical advance of the novel alum/TLR7a adjuvant.

## Extended evaluation of alum/TLR7 in relevant and challenging disease models

After the initial non-clinical proof-of-concept, the adjuvant was evaluated more extensively with a range of antigens representing pathogens for which improved or new vaccines are needed^30,42–44^. These included vaccines that are thought to require a potent T-cell induction, such as a vaccine against *Staphylococcus aureus* (SA). SA vaccines are an urgent medical need due to the sharp increase in antimicrobial resistance, but their development is hampered by a limited understanding of the protective immune mechanisms and the presence of numerous toxins, and immune evasion factors produced by the organism^45^. It is thought that Th1/Th17-polarized responses, which potentiate opsonophagocytic APC activities, may be key to an effective vaccine^43,46^.

In mouse models of SA infection, we found that two alum/TLR7a adjuvanted immunizations with conserved SA protein antigens provided 80-90% protection against challenge with a panel of SA strains^43^. Compared with an alum-only adjuvanted SA vaccine, the new adjuvant increased Th1 responses and functional (IgG2a) titers, with low Th17 responses. Follow-on studies confirmed the induction of Th1/Th17 immunity, which (in conjunction with the humoral responses) was shown to provide protection^47^. We also evaluated the adjuvant’s ability to improve a licensed alum-formulated acellular pertussis (aP) vaccine. Current aP vaccines require several childhood immunizations and boosters in adulthood^48^, but promote minimal Th1 and/or respiratory tract cellular responses. Similar to the SA vaccine model, addition of the TLR7a to an alum-absorbed aP vaccine was found to increase *B. pertussis* specific Th1/Th17 immunity, antibody functionality and IgG2a titers, which collectively enhanced protection against aerosol challenge^46^. This suggests that the adjuvant may be an effective tool for new multicomponent SA vaccines, and for improved early life protection by aP vaccines.

Proof of the adjuvant’s effectiveness with conjugate vaccines was provided by Buonsanti et al., who compared alum/TLR7a adjuvanted monovalent MenC or multivalent MenACWY polysaccharide CRM_197_-conjugate vaccines with the licensed alum-adjuvanted vaccines^42^. Following a single dose, the alum/TLR7a-immunized mice showed improved potency for Th1-skewed responses and protective anti-MenC SBA titers, which persisted at 8 months post-immunization. Similarly, responses to all antigens of the multivalent vaccine were enhanced by the SMIP-based adjuvant.

**Fig. 2:**
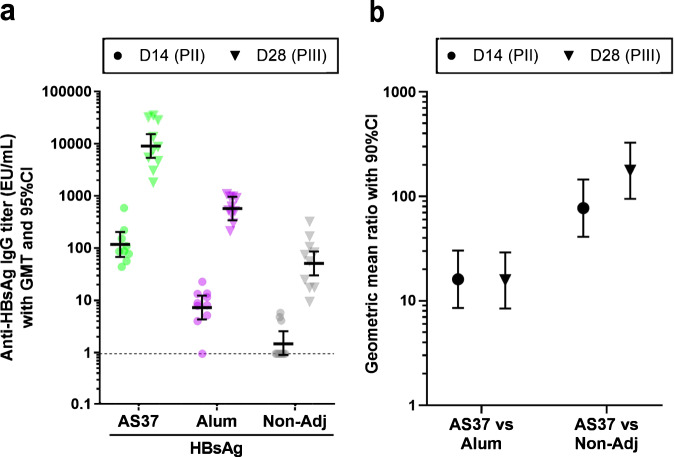
Benchmarking of the alum/TLR7a vs alum alone in mice. BALB/c mice (*n* = 10/group) were immunized at Day (D0) and D14 with hepatitis B surface antigen (HBsAg; 2 μg) vaccines that were either non-adjuvanted or formulated with either the alum/TLR7a (named AS37) or aluminum hydroxide (alum). Anti-HBsAg IgG antibody titers 14 days after the first and second vaccination (D14 and D28, respectively) are expressed in ELISA units (EU)/mL. **a** Titers are presented as individual data (symbols) and geometric means with 95% confidence intervals (CIs; bars). The dotted line represents the cutoff of 0.95 assigned to negative samples (corresponding to: LOQ × dilution/2; where the LOQ [limit of quantitation] was 50 and the dilution was 0.038). **b** An ANOVA model for repeated measures including group, timing and interaction as fixed effects was fitted on the log_10_ titers and used to compute geometric mean ratios (GMRs) with 90% CIs. Statistical superiority of AS37-adjuvanted vaccine vs alum- and non-adjuvanted vaccines was confirmed at both time points (GMRs [90% CI] D14/D28: 16.1 [8.5–30.3]/15.7 [8.5–29.1] for alum and 77.1 [41.0–145.0]/175.8 [94.9–325.8] for non-adjuvanted vaccine). This data supported the advancement toward the clinical testing of HBsAg/AS37 (NCT05561673).

As there was still a relative paucity of studies with well-characterized antigens, the alum/TLR7a was also combined with hepatitis B surface antigen (HBsAg) and compared with alum-adjuvanted and non-adjuvanted HBsAg in mice (Fig. 2). Aligned with the preceding studies, this evaluation in comparison to commercial vaccines (*Engerix-B* and *Fendrix*) showed that in terms of antigen-specific serum IgG, the alum/TLR7a outperformed alum.

The superior adjuvanticity of the new adjuvant in comparison to alum alone was subsequently confirmed with multiple viral or bacterial antigens (listed in Fig. 1; unpublished data), further highlighting the adjuvant’s potential for inducing enhanced immunity. The underlying mechanism of action was further explored by Vo et al., who demonstrated that alum/TLR7a (compared to alum only) was able to boost antigen driven B-cell proliferation and expansion of antigen specific MBCs in the dLN in mice^49^. This effect was facilitated by enhanced recruitment of naive B cells supporting the GC reaction.

### Confirming the mechanism of action in NHP

Following the mouse studies, the profiling of the adjuvant’s immune signature and mechanism of action evaluations were advanced into NHPs, a translationally more relevant model for humans. A first proof of concept of the adjuvant’s utility in this model was obtained through the induction of enhanced HIV-1 envelope glycoprotein (Env) specific B and T cell responses^50^. Next, Liang et al. described in the same model the mechanism of action by which the alum/TLR7a induced rapid cell infiltration to the muscle between 24 and 72 h post-injection, resulting in more antigen-loaded neutrophils, monocytes and DCs migrating to the dLN, relative to alum alone^33^. The ensuing increased Tfh and GC responses were shown to result in type I IFN induction, and potent Th1 responses.

The adjuvant was also included in a comparative study by Francica et al., along with several alternative adjuvants (including alum alone, alum/TLR4a, and oil-in-water emulsions with/without TLR4a or the TLR7a)^44^ to allow ‘benchmarking’. Through asking how innate activation alters the HIV Env-specific response, the authors observed distinct transcriptional signatures for alum/TLR4a *vs* alum/TLR7a. The expression of inflammatory genes was upregulated for the TLR4a, while it was suppressed for alum/TLR7a, which preferentially upregulated antiviral and IFN gene expression. Both the alum/TLR4a and alum/TLR7a boosted the binding antibody titers compared with alum only, but the boost was approximately three-fold stronger for the alum/TLR7a adjuvant.

Recently, a Th1-biased response to the AS37 adjuvant was also observed in preclinical SARS-CoV-2 studies, first in mice^51^ and then in a comparative adjuvant study in NHPs^52^. Early immune signatures in both models differed for the TLRa-based adjuvants (AS37 and CpG1018) versus other adjuvants (alum, AS03, or another emulsion-based adjuvant), resulting in mice in Th1-skewed CD4^+^ T cell responses versus a balanced Th1/Th2 response, respectively. Although the AS37-mediated Th1 cytokine response seen in mice was not observed in NHPs in this study, this may be explained by the known difficulties in extrapolating T cell response patterns from murine to simian models^53^ and may reflect the use of a novel engineered nanoparticle antigen. Nevertheless, the immunity promoted by AS37, AS03, and the TLR9a offered in both models a long-term protection against viral challenge^51,52^. Overall, this data confirmed that activation of the TLR7 pathway was consistent between rodents and NHPs. The comparative adjuvant studies helped to position the new TLR7a based adjuvant within a wide range of adjuvant options, highlighting its potential use in vaccines against more challenging pathogens.

### Toxicology evaluations

In advance of the first in-human evaluation, the adjuvant’s preclinical safety profile was evaluated through a range of toxicology studies (unpublished data). In vitro screening indicated the absence of phototoxicity, genotoxicity and mutagenic effects. By i.m. route, alum/TLR7a was well tolerated in rats, dogs and NHPs. This was corroborated by its PK profile (in rats/dogs) showing that localization was restricted to the injection site, without local toxicity. Systemic exposure was minimal, and the observed effects were limited to non-adverse changes in clinical pathology/histopathology, indicative of inflammation, consistent with TLR7a activity. The initial murine studies had showed that TLR7a adsorption onto alum balanced the blood cytokine levels *vs* the unformulated molecule, while improving immunogenicity^30^. Moreover, no specific gene activation was observed in splenocytes from alum/TLR7a-treated TLR7-knock out mice^42^, suggesting that the adjuvanticity was—in contrast to resiquimod^29^—fully TLR7-dependent without any off-target activity.

## Human safety and cross-species Th1-mediated mechanism of action

With safety as the primary endpoint, but including immunogenicity endpoints in the protocol, the adjuvant (AS37) was evaluated in a Phase 1 dose escalation study in healthy adults (TLR7a doses: 12.5, 25, 50, or 100 µg)^54^. In this study, AS37 was combined with MenC-CRM_197_ conjugate antigen, which provides a suitable control, since it is licensed with an extensive safety database established from long-term use^55^. While a single dose of MenC is recommended for persons over 1 year of age, three doses are recommended for infant immunization for which the adjuvant might offer dose sparing opportunities. Hence the TLR7a was evaluated in a dose-escalating fashion, with a fixed dose of alum, that was consistent with the dose used in the licensed vaccine^54^.

In this study, the investigational vaccine formulations containing AS37 with TLR7a dose 12.5–50 µg showed acceptable safety profiles, with no reports of severe local AEs in these groups and just one report of severe systemic AE, in the AS37-25µg group (6.3%). However, in the AS37-100µg group there were three reports of severe solicited systemic AEs (18.8%) and one report of severe local AE (6.3%), although additional studies would be helpful in clarifying the safety profile of a vaccine containing the highest dose of TLR7a. Overall, the number of participants experiencing unsolicited AEs was comparable between the study vaccine groups and the control MenC-CRM_197_ group. No clinically-significant abnormal laboratory values or AE-related withdrawals from the study were reported^54^. Plasma TLR7a levels slightly rose with increasing adjuvant doses, but remained low (geometric mean concentrations <100 ng/L). This suggested only limited systemic exposure, as expected from the nonclinical data. Compared to the control vaccine, no increase in the frequency of any solicited systemic AEs or unsolicited AE was reported in the AS37-adjuvanted groups. However, the authors could not exclude the possibility that the TLR7a plasma level may have contributed to the higher frequencies of severe solicited systemic AEs observed in the group with the 100 µg TLR7a dose.

Similar to the absence of a clear dose-dependent reactogenicity profile in the formulations with the three lower doses of TLR7a, dose-dependent responses in the antibody titers were inconsistently observed across readouts^34^. While geometric mean titers of MenC polysaccharide binding antibody responses were doubled in the two higher-dose cohorts *vs* the remaining groups—and thus tended to be dose-dependent– hSBA titers were similar across cohorts. Peripheral CRM_197_-specific Th1, Th2 and Th17 responses to vaccination were similarly increased across groups, but the Tfh responses over pre-vaccination baseline were highest in the AS37 cohorts. The groups immunized with the three highest AS37 doses also displayed the highest MenC-specific MBC responses, which may reflect TLR7-mediated effects on Tfh cells via crosstalk with DCs and B cells. Several explanations for the overall limited adjuvanticity with this vaccine in this population were proposed^34^, including the presence of alum in both the control and the test groups, the already robust immunogenicity of the control vaccine in healthy young adults^55,56^, and given the high MenC prevalence in the study location in the years preceding the trial^57^, the memory responses most likely present in most individuals at baseline.

In contrast to these modest effects on the adaptive response, the early cytokine, transcriptional, and innate cell responses were strongly enhanced in the three days after a single injection with AS37^34^. Of the 30 serum cytokines evaluated, the IFN-γ-inducible chemokine IP-10 displayed increased levels specifically in the AS37 groups, aligned with concurrent CXCL10 mRNA responses in blood. The omics data revealed that AS37 induced systemic, transient (<1-week) activation of the IFN-mediated antiviral response in a dose-dependent manner up to 50 μg TLR7A, with a lower effect for high dose TLR7a. Genes involved in metabolic processes, protein production and B-cell responses were also upregulated, though the latter were at similar levels for alum and AS37. By contrast, Day 1 IFN responses were only seen with AS37. This was supported by an integrated data analysis of TLR7a-specific effects, which confirmed the IFN-mediated response and increased APC activation. Correspondingly, activated pDCs and intermediate monocyte responses were elevated in AS37 recipients, particularly with the two higher doses. Hence, the new adjuvant appeared to operate in man as intended, even though the Men C vaccine did not appear to significantly benefit from an improved adjuvant in this patient population.

Overall, these clinical data supports a model in which AS37 promoted activation of pDCs and upregulated both the expression of antiviral/IFN-inducible genes and IFN production by lymphocytes or monocytes, which in turn triggered systemic CXCL10/IP-10 responses and innate cell recruitment. The transient innate immune activation peaked at 24 h and resolved within 7 days—aligned with the kinetics seen for the other AS seen in human blood^4,6^—and finally evolved into potent Tfh, MBC and antibody responses. The cross species similarities between humans and non-human primates, that were found irrespective of the antigen, supported the notion that the immune signature of AS37 is consistent with the hallmark of TLR7 engagement, inducing an antiviral state through activation of the NF-κB pathway and type-1 IFN secretion by pDCs^22,58^.

### Clinical adjuvant benchmarking

Based on the data from the first clinical study of TLR7a the potential of AS37 versus alternative adjuvants remained ill-defined, as we had no insights on the translatability of data on protein/polysacharide conjugates *vs* recombinant proteins, and on heavily antigen-primed *vs* naive populations. Previously, the adjuvant effect of alum, AS01 (containing MPL, QS-21 and liposomes), AS03, and AS04 had been compared clinically with several subunit antigens^1,2^, most notably with HBsAg in a series of translational science studies^4,6–9^. Indeed, from a single pivotal trial in HBsAg-naive adults, extensive comparisons for alternative adjuvants could be done, which included serum cytokines, innate and adaptive cells, transcriptomics, and antibody avidity, persistence and Fc-mediated features, with more data to follow. Encouraged by this data, a Phase I/IIa study of different adjuvanted HBsAg vaccines was initiated in antigen-naive adults (NCT05561673). The study comprises a head-to-head comparison of AS03, AS04, alum, and two formulations of AS37. The study aims to compare the safety, reactogenicity and adjuvanticity of these formulations, in order to establish the clinical utility and positioning of AS37 as a novel adjuvant candidate.

## Conclusions and perspectives

The elucidation of the mechanisms of TLR7 activation and the flexibility inherent to the SMIP approach enabled a rational design of a BZN TLR7a SMIP. The synthetic molecule was modified with PEG linkers (allowing for increased solubility at neutral pH) and an anionic terminal phosphonate group to allow robust adsorption to alum by ligand exchange, yielding the AS37 adjuvant candidate. In multiple animal models, it was demonstrated that this physicochemical modification allowed the alum adsorbed SMIP to mediate robust APC activation and priming of Th1 cells in the dLN, leading to quantitative and qualitative enhancement of Th1-skewed cell mediated and humoral responses against various pathogen based antigens. Systemic dissemination of the SMIP was limited, reflected by the minimal side effects in humans within the main dose range evaluated. Finally, these modifications also facilitated industrial scale-up and manufacturing of liquid, fridge stable vaccine candidates.

Several alternative TLR7a molecules and delivery systems (e.g. encapsulation in nanoparticles or conjugation to polymers) have emerged in the intervening decade since we described the discovery of SMIPs (reviewed in ref. ^29^), as have mixtures of TLR7a with other TLRa molecules^59^. Some of these adjuvants have entered clinical development. For example, different formulations of an imidazoquinoline-based molecule bearing a fatty-acid tail (3M-052^60^) have entered clinical trials in the context of an HIV-1 candidate vaccine^61^. The broad TLR7-mediated immune stimulation by the adjuvant described here and by other TLR7a-based adjuvants has been exploited in the context of COVID-19^38,51,52,62–66^, with an example of the whole-virion SARS-Cov-2 vaccine containing an alum-adsorbed TLR7/8a adjuvant^14,67,68^. The latter vaccine, licensed in India in 2020 and distributed for emergency use^69^, has currently been administered to over 313 million people in India alone^14^. It was shown to be well tolerated with 77.8%/93.4% efficacy for symptomatic/severe symptomatic disease (interim Phase III data)^14^. This, as well as murine data in the context of influenza^70^, suggests that an alum/TLR7a is also effective in whole virion vaccines, which are traditionally alum-adjuvanted and generate mostly Th2-biased responses. Ultimately, the comparisons with the clinically established AS in an ongoing trial will help to determine the role of AS37 in future clinical vaccine development.

Overall, the success of clinically advanced alum-adsorbed TLR7 adjuvants, notably in the context of SARS-CoV-2, have shown their potential in enhancing Th1-biased response, which may help overcome hyporesponsiveness in certain populations. Pending its additional characterization and positioning in the larger adjuvant portfolio, AS37 is a promising asset for use in next generation vaccines targeting infectious diseases.

## Data Availability

The data presented in Fig. 2 are available from the authors upon request. All other data included in this review were obtained from publicly available sources and cited accordingly. The references for each study or publication included in this review are provided for further reading and verification.
